# Paraneoplastic dermatomyositis-like syndrome preceding diagnosis of small cell lung cancer: a case report

**DOI:** 10.3389/fonc.2026.1915555

**Published:** 2026-07-31

**Authors:** Jinwei Wang, Meiling Jiang, Kun Chen, Bo Zhang, Yan Xiao

**Affiliations:** Beijing GoBroad Hospital, Beijing, China

**Keywords:** cross reaction, dermatomyositis, erythroderma, paraneoplastic syndrome, small cell lung cancer

## Abstract

Paraneoplastic syndromes (PNS) are frequently misdiagnosed as independent neurological, endocrine, cutaneous, or rheumatic immune disorders due to their low incidence, highly variable, and complex clinical presentations. Currently, when faced with clinically unexplainable phenomena, PNS is seldom considered in the differential diagnosis. A 68-year-old non-smoking Chinese male presented with insidious digital clubbing in 2021 and refractory cutaneous eruptions in 2023, which were initially diagnosed as granulomatous rosacea and erythroderma. However, treatment with ketotifen, cetirizine, and the immunosuppressant cyclosporine led only to partial regression of the rash, with persistent pruritus. Approximately 3 years after the insidious onset of digital clubbing and 1 year after the skin lesions flared, in April 2024, chest PET/CT scan revealed a mass in the left lower lobe and a nodule in the dorsal segment of the right lower lobe. A biopsy confirmed extensive-stage small cell lung carcinoma (SCLC).His dermatological manifestations, which progressed to edema and dysphagia, and ancillary examination results were subsequently attributed to a paraneoplastic dermatomyositis-like syndrome. Chemotherapy for SCLC resulted in resolution of the skin lesions and disease control. This case underscores the critical role of PNS as an early indicator of occult malignancy and highlights the therapeutic challenges in managing paraneoplastic dermatomyositis-like syndrome.

## Introduction

Paraneoplastic syndromes (PNS) occur in approximately 10% to 20% of all malignancies, with small cell lung cancer (SCLC) being one of the neoplasms most frequently associated with paraneoplastic phenomena (1). In SCLC specifically, paraneoplastic manifestations affect up to 5% of cases and encompass a broad spectrum of disorders, including the syndrome of inappropriate antidiuretic hormone secretion, Cushing syndrome, Lambert-Eaton myasthenic syndrome, paraneoplastic cerebellar degeneration, and various cutaneous and musculoskeletal syndromes (2). Among the endocrine PNS, ectopic hormone production is particularly common in SCLC, and these syndromes may precede or signal recurrence of the primary malignancy (3). Beyond neurologic and endocrine manifestations, cutaneous paraneoplastic syndromes are increasingly recognized as important early indicators of occult malignancy (4), often presenting before the cancer diagnosis becomes clinically apparent.

Among dermatologic paraneoplastic syndromes associated with small cell lung cancer, dermatomyositis (DM) and digital clubbing/hypertrophic osteoarthropathy (HOA) are the most diagnostically challenging. Approximately 20% of adult DM cases are paraneoplastic, with small cell lung cancer being a common associated malignancy; about one third of all DM cases are malignancy related, particularly within three years of diagnosis (5). Classic DM and amyopathic DM carry similarly elevated malignancy risk. Importantly, DM may precede cancer diagnosis by months to two years, offering a critical window for early detection (6). Similarly, Digital clubbing, often a feature of hypertrophic osteoarthropathy (HOA), is a paraneoplastic manifestation of SCLC. HOA is characterized by clubbing, periostitis, and arthralgia, and is associated with malignancy in up to 90% of adult cases; pulmonary tumors account for ~80% of these, though predominantly non-small cell types. Isolated clubbing occurs in ~20% of SCLC patients. Like dermatomyositis, HOA can precede cancer diagnosis by months, highlighting the value of early recognition (7).

Despite known associations, paraneoplastic syndromes (PNS) are often underdiagnosed. In a real-world cohort of >46,000 SCLC patients, Lambert-Eaton myasthenic syndrome (LEMS) was diagnosed in only 0.16% versus the expected 3–6%, highlighting marked under recognition (8). This diagnostic gap likely extends to cutaneous and musculoskeletal PNS as well, where skin manifestations may be misdiagnosed as primary inflammatory disorders, leading to delays in cancer detection and suboptimal outcomes.

We report a case of a 68-year old non-smoking Chinese male who presented with insidious digital clubbing in 2021 followed by refractory cutaneous eruptions, both of which were initially managed as independent dermatologic conditions. An underlying extensive-stage SCLC was identified about 3 years after the insidious onset of digital clubbing and 1 year after the skin lesions flared. The dermatologic manifestations, which progressed to edema and dysphagia, were later recognized as paraneoplastic. This case underscores the critical importance of maintaining a high index of suspicion for PNS in patients presenting with treatment resistant dermatoses or unexplained clubbing, even in the absence of respiratory symptoms, and highlights the need for heightened malignancy surveillance in such patients.

## Case presentation

**Figure 1:**
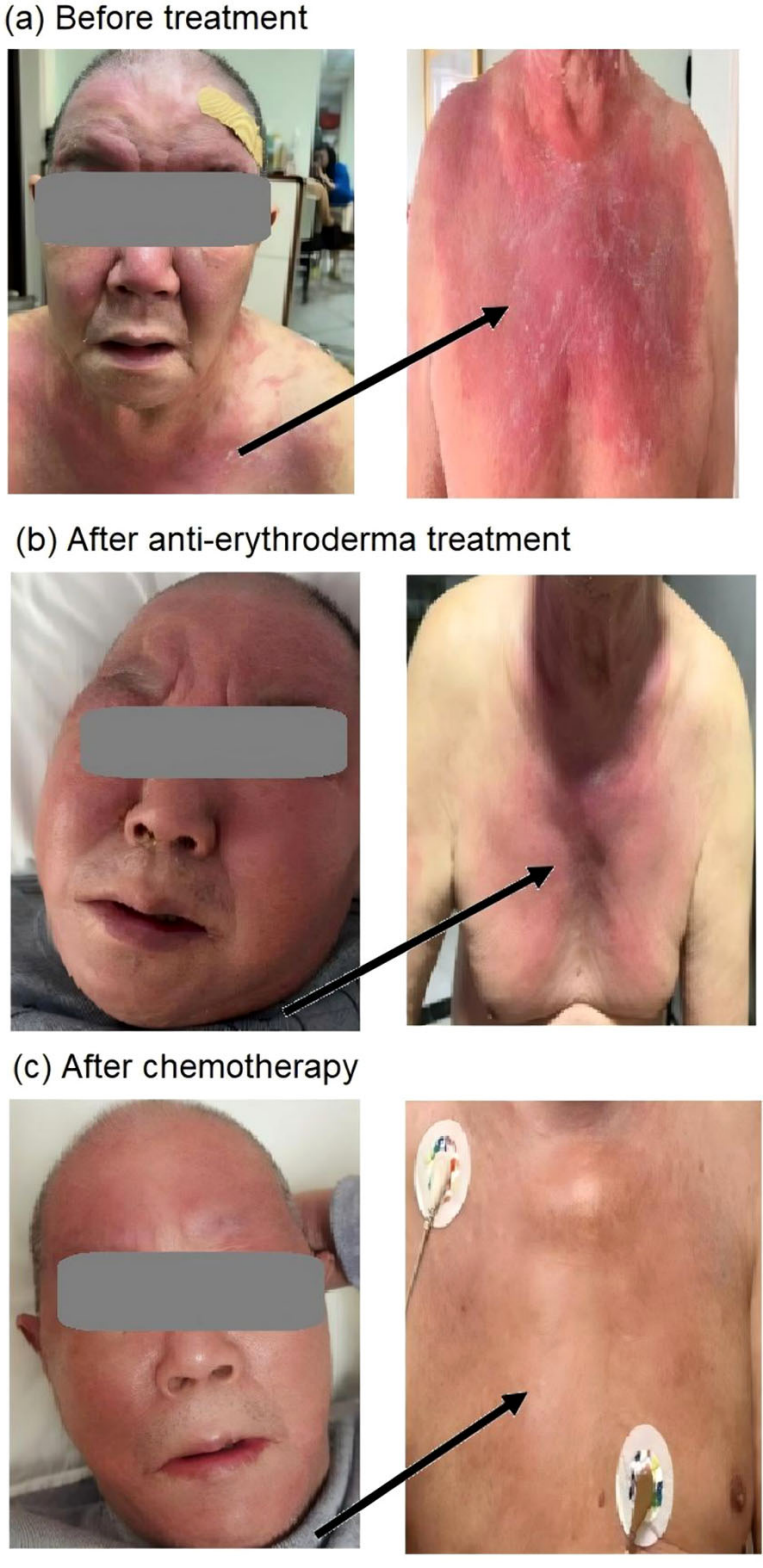
**(a)** Picture of the patient at presentation. Before treatment, erythema affected most of the body skin surface, with scaling and pruritus. **(b)** Picture after anti- erythroderma treatment (antihistamines and cyclosporine), showing partial resolution of the rash but persistent pruritus. **(c)** Picture after chemotherapy (etoposide-platinum followed by paclitaxel); the erythema resolved completely.

A 68-year-old male, with no smoking or family history of malignancy, developed painless digital clubbing in 2021 without respiratory or joint symptoms and did not seek medical attention. In 2023, he gradually developed facial erythema and edema, progressing to a generalized pruritic rash involving the head, neck, trunk, and extremities (**Figure 1a**), without muscle weakness or fever. Topical calamine was ineffective; antihistamines (ketotifen, cetirizine) and cyclosporine achieved partial rash resolution but pruritus persisted (**Figure 1b**).

By early 2024, he developed progressive facial and extremity edema with mild solid dysphagia. Forehead skin biopsy revealed mild epidermal atrophy, scattered apoptotic cells, multifocal basal cell degeneration, dermal edema, and increased mucin deposition, consistent with interface dermatitis, perivascular lymphocytic infiltration, and mucin deposition. IHC showed a mixed lymphocytic infiltrate (CD3+, CD4+, CD8+, focally CD20+, CD163+) (**Supplementary Figure 1**). The clinical diagnosis was erythroderma.

**Figure 2:**
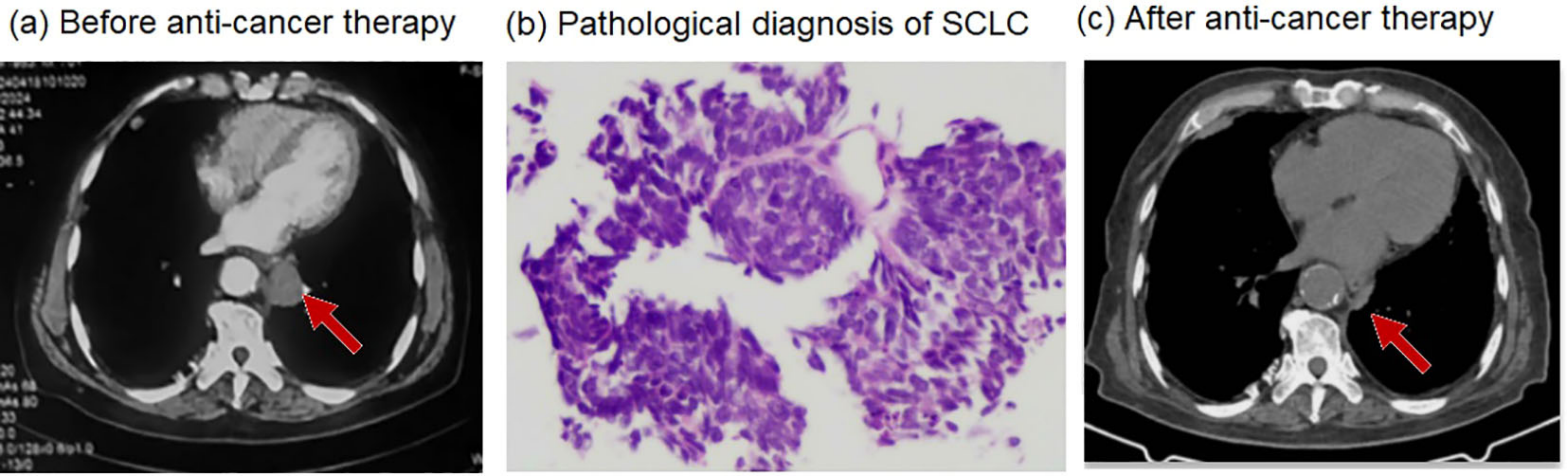
**(a)** Chest PET/CT showing nodular pleural thickening, mediastinal lymphadenopathy, and a soft tissue mass adjacent to the spine in the left lower lobe of the lung. **(b)** Biopsy histology (hematoxylin & eosin, ×20) showing atypical cells with scanty cytoplasm arranged in a diffuse, sheet-like or nested pattern, consistent with small cell lung carcinoma. **(c)** Chest PET/CT after treatment showing complete disappearance of the tumor (red arrow) - complete remission.

In April 2024, approximately 3 years after clubbing onset and 1 year after rash flare, chest PET/CT revealed nodular pleural thickening, mediastinal lymphadenopathy, and a left lower lobe paravertebral soft tissue mass (**Figure 2a**), measuring ~5.0×2.8×2.4 cm with intense uptake (SUVmax 19.0). A right lower lobe dorsal segment nodule (~1.3×1.1 cm, SUVmax 7.5) was also noted (**Supplementary Figure 2**). Tumor biopsy of the left lower lobe mass showed atypical cells with scant cytoplasm arranged in sheets or nests, consistent with SCLC (**Figure 2b**). IHC revealed: CK (pancytokeratin) diffuse positive (+); CgA (+); Ki-67 (~80%+); TTF-1 (diffuse+); INSM1 (majority+); P40 (−); CK5/6 (occasional); Napsin A (−); NUT (−); BRG1 (diffuse+) (**Supplementary Figure 3**). These findings confirmed SCLC, staged as extensive-stage based on contralateral pulmonary metastasis and bilateral mediastinal/hilar lymphadenopathy.

To investigate the paraneoplastic nature of the skin manifestations, further workup in May 2024 showed markedly elevated CK (1,281 U/L; reference 25–190 U/L) (**Supplementary Figure 4**), shortened motor unit potential durations on EMG (**Supplementary Figure 5**), and borderline positivity for anti-TIF1γ, anti-OJ, and anti-SRP, with weak anti-Th/To positivity (**Supplementary Figure 6**). Collectively, these findings supported a dermatomyositis-like paraneoplastic neurological syndrome.

From May to October 2024, the patient received 7 cycles of first-line etoposide plus platinum with trilaciclib (240 mg/m^2^, to mitigate myelotoxicity and transfusion needs) achieving a partial response (PR), accompanied by complete resolution of rash and pruritus (**Figure 1c**). In April 2025, based on confirmed disease progression (a 25% increase in target lesion size per RECIST 1.1) and worsening skin rash,the regimen was switched to second-line nab-paclitaxel monotherapy. We note that topotecan is also a standard option for extensive-stage SCLC, we opted for nab-paclitaxel in this patient because of his advanced age and poor performance status. We have clarified this in the revised manuscript. After two cycles of nab-paclitaxel treatment, follow-up imaging confirmed a complete response (CR) (**Figure 2c**) and resolution of rash and pruritus. The final diagnosis was paraneoplastic dermatomyositis-like syndrome in the setting of SCLC. Unfortunately, the complete remission period was brief (~3 months). Tumor and skin rash progressed from August 2025 onward. In November 2025, liver metastases appeared, tumor markers rose significantly, and the patient died of liver failure in the same month. Clinical timeline of the patient`s disease course was shown in **Supplementary Table 1**.

## Discussion

In the present case, the initial manifestations were digital clubbing (in 2021) and a refractory skin rash (in 2023), appearing 1–3 years prior to the eventual diagnosis of lung cancer. This temporal sequence is consistent with the well-recognized “prodromal” feature of paraneoplastic syndromes (PNS), in which cutaneous or rheumatologic signs often precede the detection of the underlying malignancy by months to several years (9). Early skin biopsy findings were initially misdiagnosed as granulomatous rosacea and erythroderma. Several factors likely contributed to this diagnostic delay. First, PNS related skin lesions lack specific histopathological markers, making it difficult to distinguish them from primary inflammatory dermatoses. Second, at the time of initial presentation, a comprehensive systemic screening for occult malignancy (such as lowdose chest CT or PET/CT) was not performed, reflecting a low index of suspicion for PNS in the absence of overt respiratory or constitutional symptoms. This case therefore reinforces the critical importance of including PNS in the differential diagnosis when encountering atypical, treatment resistant dermatological conditions, even in patients without any obvious cancer related symptoms.

A particularly instructive observation in this case was the temporal correlation between tumor response and skin rash improvement. Following chemotherapy, the patient achieved partial remission of the small cell lung carcinoma (SCLC), accompanied by significant resolution of the cutaneous lesions. This parallel evolution, improvement of paraneoplastic manifestations with effective antineoplastic therapy, strongly supports the paraneoplastic nature of the dermatological findings. Such a dynamic relationship has been consistently documented in the literature; for instance, Guan et al. reported a SCLC patient whose dermatomyositis symptoms notably ameliorated as the malignancy regressed after two cycles of chemotherapy. In that case, the authors emphasized that diligent malignancy screening is imperative for patients diagnosed with dermatomyositis (10). Different reports showed that SCLC is associated with paraneoplastic syndromes, including dermatomyositis, and that cancer-associated dermatomyositis (CAD) commonly presents in conjunction with SCLC (11). Conversely, before the SCLC diagnosis, our patient had received immunosuppressive agents including cyclosporine for the presumed primary dermatoses. Although these agents provided partial rash control, their use in the setting of an occult malignancy raises a theoretical concern: immunosuppression may inadvertently facilitate tumor progression by attenuating antitumor immune surveillance as reported by other group (12). This concern underscores the need for early differential diagnosis to avoid prolonged empirical immunosuppression in patients who may actually have a paraneoplastic process requiring oncologic management.

For the treatment selection of this patient, the current standard of care for extensive-stage small cell lung cancer (ES-SCLC), as recommended by major international guidelines, is a combination of etoposide–carboplatin (EC) chemotherapy plus immune checkpoint inhibitors (ICI). However, reports on the use of ICI for cancer-associated dermatomyositis remain conflicting (11). Given our patient`s pre-existing pulmonary fibrosis, ICI was considered contraindicated; we therefore opted for conventional EC chemotherapy alone as the first line treatment. Regarding the mechanism of the favorable second-line response, we suggest that nab-paclitaxel, unlike platinum-based DNA-damaging agents, acts via microtubule stabilization and may induce ferroptosis—a cell death pathway particularly active in SCLC (13)—thereby bypassing first-line resistance. Complete remission with nab-paclitaxel monotherapy in refractory SCLC has also been reported (14). However, due to adverse effects, only two cycles were given, and the response was short-lived. Whether additional mechanisms exist beyond ferroptosis requires further study.

Regarding the mechanistic link between SCLC and the dermatomyositis-like presentation observed in this case, it is plausible that SCLC expresses neuroendocrine associated antigens (such as anti-Hu antibodies or ANNA1) that cross react with self antigens in skin and muscle tissues, thereby triggering an autoimmune mediated dermatomyositis phenotype (15). Emerging evidence has identified anti-transcription intermediary factor 1gamma (TIF1γ) as a highly specific autoantibody for cancer-associated dermatomyositis (16). Arnon et al. described a SCLC patient with paraneoplastic dermatomyositis-like syndrome who tested positive for anti-TIF1γ and was found to harbor a point mutation in the TIF1γ coding gene, suggesting a direct pathogenic role of the autoantibody through immune cross reactivity (17).Our patient exhibited borderline positivity for anti-TIF1γ, anti-OJ, and anti-SRP, along with weak anti-Th/To positivity (**Supplementary Figure 6**), which consistent with the cross-reactivity hypothesis.

## Conclusions

In summary, this case report delivers several clinically actionable messages. First, new onset digital clubbing in adults, particularly when accompanied by atypical or refractory skin lesions, should be regarded as a “red flag” for an underlying malignancy, even in the absence of respiratory symptoms. Second, for skin lesions suspected to be paraneoplastic in origin, a thorough diagnostic workup should be initiated early, including systemic imaging studies such as low dose chest CT or whole body PET/CT, as well as serum tumor marker screening. Third, the parallel response of paraneoplastic skin lesions to effective cancer therapy not only confirms the diagnosis but also serves as a useful clinical biomarker of treatment efficacy. Finally, raising awareness of PNS among clinicians across multiple specialties (dermatology, rheumatology, pulmonology, and oncology) is essential to reduce diagnostic delays and improve patient outcomes. This case adds to the growing body of evidence that cutaneous PNS are not merely rare curiosities but rather important early sentinel signs of occult malignancy that, when recognized promptly, can lead to earlier diagnosis and potentially life prolonging treatment.

## Data Availability

The original contributions presented in the study are included in the article/**Supplementary Material**. Further inquiries can be directed to the corresponding authors.
