# Supplementary material for: Paraneoplastic dermatomyositis-like syndrome preceding diagnosis of small cell lung cancer: a case report
Source: Front Oncol. 2026 Jul 31;16:1915555. doi: 10.3389/fonc.2026.1915555 (PMC13472917; doi:10.3389/fonc.2026.1915555)
Supplement: Supplementary file 1 [file DataSheet1.pdf]

Supplemental data

Supplemental Table 1

Time line	Events	Remarks
2021	Digital clubbing	Initial paraneoplastic manifestation
2023	Skin rash	Progressive cutaneous involvement
Apr 2024	Forehead skin biopsy (pathology & IHC)	Erythroderma (Interface dermatitis with mucin deposition & mixed lymphocytic infiltration)
Apr 2024	PET/CT	Lung mass detected (Extensive-stage)
Apr 2024	Tumor biopsy (pathology & IHC)	SCLC
May 2024	Serological and EMG tests	DM-like changes
May 2024 - Oct 2024	First-line chemotherapy	EC
Follow-up	Imaging	Confirmed PR on follow-up imaging
	Clinical	Resolution of DM- like features (Fig.1c)
Apr 2025 - May 2025	Switch to second-line (nab-paclitaxel monotherapy)	Due to the progression of the tumor and skin rash
Follow-up	Imaging	Brief complete remission (~3 months) (Fig.2c) then progression from Aug. 2025 onward
	Clinical	Resolution of skin rash, then progression; died of hepatic metastases and liver failure in Nov. 2025

Supplemental Table 1. Clinical timeline of the patient‘s disease course. This timeline illustrates the sequential clinical events from initial paraneoplastic manifestations to diagnosis, treatment, and final outcome.

Abbreviations: SCLC, small cell lung carcinoma; DM, dermatomyositis; EMG, electromyography; CK, creatine kinase; PR, partial response; CR, complete response; EC, etoposide plus carboplatin.

Supplemental Fig.1

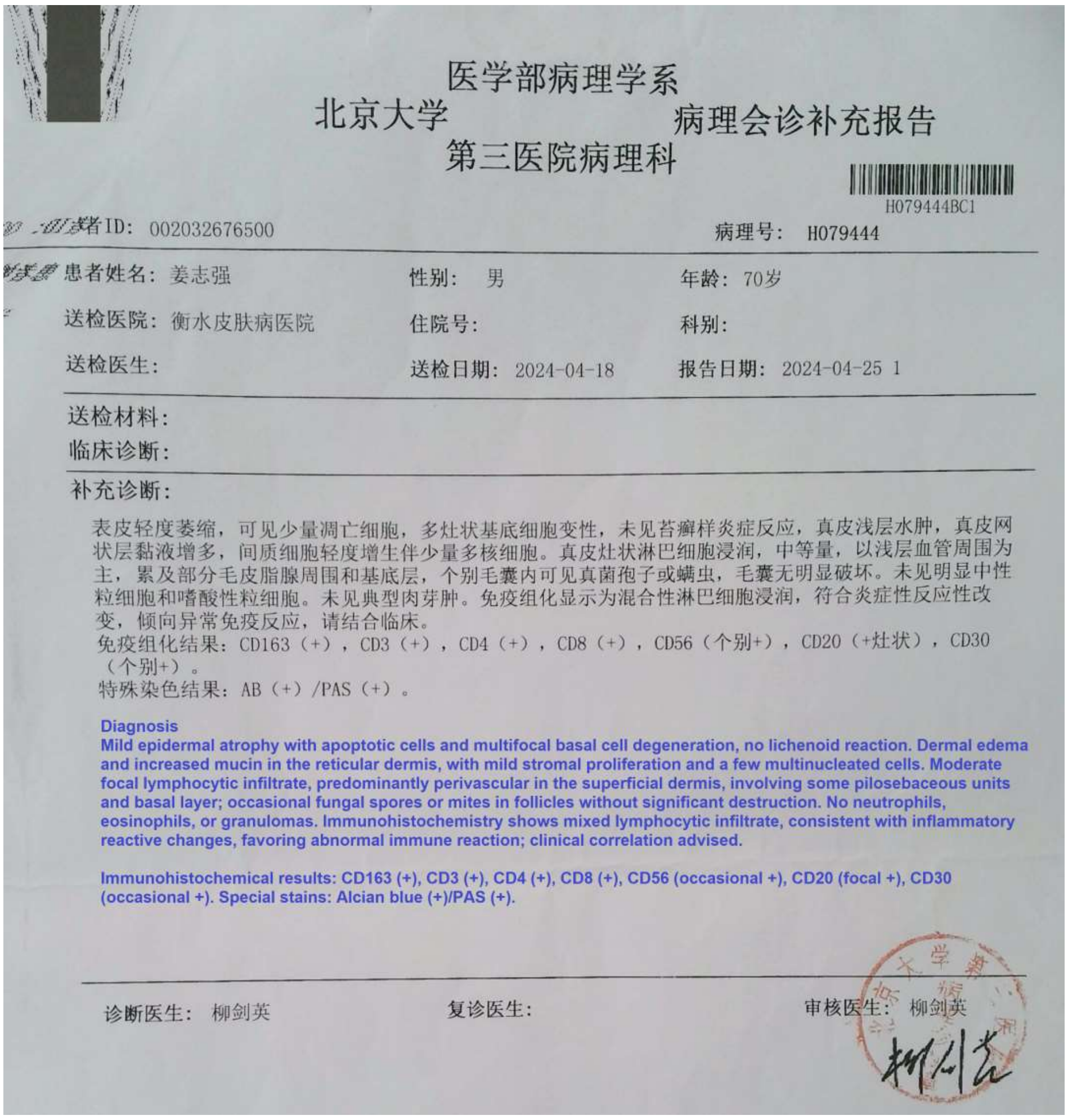

[redacted]

医学部病理学系
北京大学 病理会诊补充报告
第三医院病理科

H079444BC1

者ID: 002032676500 病理号: H079444

患者姓名: 姜志强 性别: 男 年龄: 70岁

送检医院: 衡水皮肤病医院 住院号: 科别:

送检医生: 送检日期: 2024-04-18 报告日期: 2024-04-25 1

送检材料:

临床诊断:

补充诊断:

表皮轻度萎缩，可见少量凋亡细胞，多灶状基底细胞变性，未见苔癣样炎症反应，真皮浅层水肿，真皮网状层黏液增多，间质细胞轻度增生伴少量多核细胞。真皮灶状淋巴细胞浸润，中等量，以浅层血管周围为主，累及部分毛皮脂腺周围和基底层，个别毛囊内可见真菌孢子或螨虫，毛囊无明显破坏。未见明显中性粒细胞和嗜酸性粒细胞。未见典型肉芽肿。免疫组化显示为混合性淋巴细胞浸润，符合炎症性反应性改变，倾向异常免疫反应，请结合临床。

免疫组化结果：CD163（+），CD3（+），CD4（+），CD8（+），CD56（个别+），CD20（+灶状），CD30（个别+）。

特殊染色结果：AB（+）/PAS（+）。

Diagnosis

Mild epidermal atrophy with apoptotic cells and multifocal basal cell degeneration, no lichenoid reaction. Dermal edema and increased mucin in the reticular dermis, with mild stromal proliferation and a few multinucleated cells. Moderate focal lymphocytic infiltrate, predominantly perivascular in the superficial dermis, involving some pilosebaceous units and basal layer; occasional fungal spores or mites in follicles without significant destruction. No neutrophils, eosinophils, or granulomas. Immunohistochemistry shows mixed lymphocytic infiltrate, consistent with inflammatory reactive changes, favoring abnormal immune reaction; clinical correlation advised.

Immunohistochemical results: CD163 (+), CD3 (+), CD4 (+), CD8 (+), CD56 (occasional +), CD20 (focal +), CD30 (occasional +). Special stains: Alcian blue (+)/PAS (+).

诊断医生: 柳剑英 复诊医生: 审核医生: 柳剑英

Supplemental Fig.1:Forehead skin biopsy revealed mild epidermal atrophy, scattered apoptotic cells, multifocal basal cell degeneration, dermal edema, and increased mucin deposition, consistent with interface dermatitis, perivascular lymphocytic infiltration, and mucin deposition. IHC showed a mixed lymphocytic infiltrate (CD3+, CD4+, CD8+, focally CD20+, CD163+)

Supplemental Fig.2

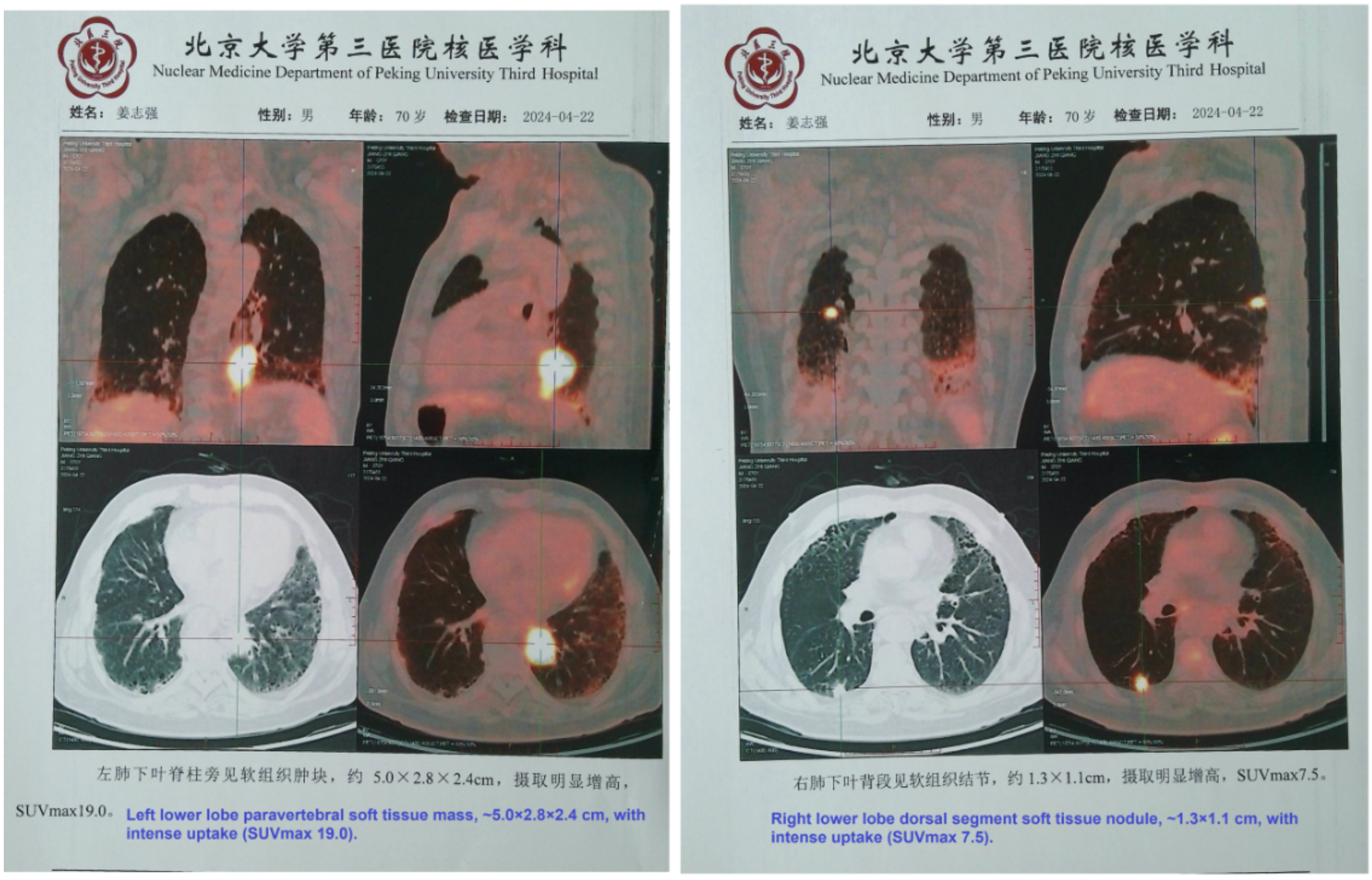

Supplemental Fig.2: Diagnosis of extensive-stage of lung cancer.

Supplemental Fig.3

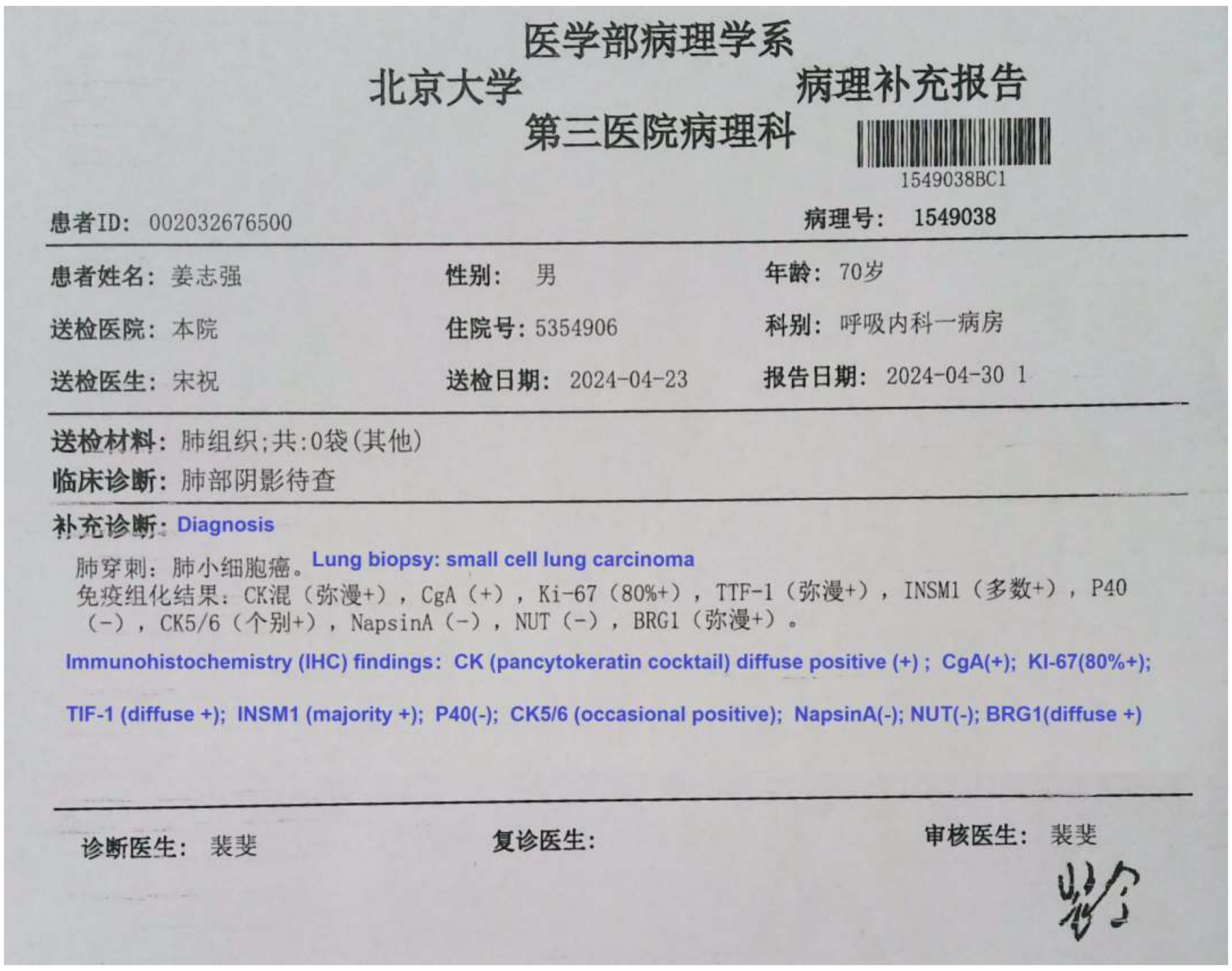

北京大学 医学部病理学系 第三医院病理科 病理补充报告

1549038BC1

患者ID：002032676500 病理号：1549038

患者姓名：姜志强 性别：男 年龄：70岁

送检医院：本院 住院号：5354906 科别：呼吸内科一病房

送检医生：宋祝 送检日期：2024-04-23 报告日期：2024-04-30 1

送检材料：肺组织;共:0袋(其他)

临床诊断：肺部阴影待查

补充诊断：Diagnosis

肺穿刺：肺小细胞癌。Lung biopsy: small cell lung carcinoma

免疫组化结果：CK混（弥漫+），CgA（+），Ki-67（80%+），TTF-1（弥漫+），INSM1（多数+），P40（-），CK5/6（个别+），NapsinA（-），NUT（-），BRG1（弥漫+）。

Immunohistochemistry (IHC) findings: CK (pancytokeratin cocktail) diffuse positive (+); CgA(+); KI-67(80%+); TIF-1 (diffuse +); INSM1 (majority +); P40(-); CK5/6 (occasional positive); NapsinA(-); NUT(-); BRG1(diffuse +)

诊断医生：裴斐 复诊医生： 审核医生：裴斐

Supplemental Fig.3: Diagnosis of SCLC by Immunohistochemistry : CK (pancytokeratin cocktail) diffuse positive (+); CgA(+); KI-67(80%+); TIF-1 (diffuse +); INSM1 (majority +); P40(-); CK5/6 (occasional positive); NapsinA(-); NUT(-); BRG1(diffuse +)

Supplemental Fig.4

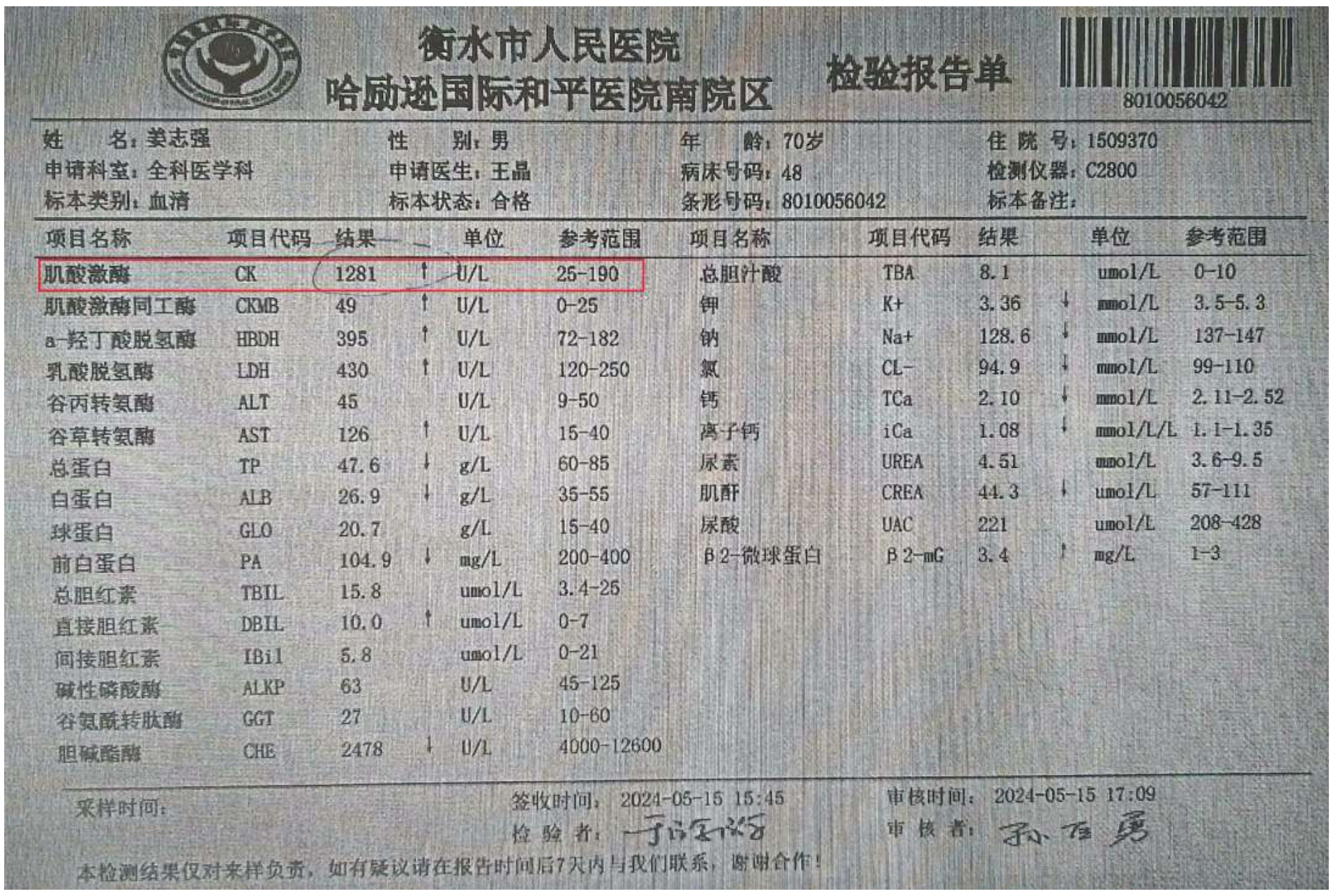

衡水市人民医院
哈励逊国际和平医院南院区
检验报告单
8010056042

姓　名：姜志强　性　别：男　年　龄：70岁　住 院 号：1509370
申请科室：全科医学科　申请医生：王晶　病床号码：48　检测仪器：C2800
标本类别：血清　标本状态：合格　条形号码：8010056042　标本备注：

项目名称	项目代码	结果	单位	参考范围	项目名称	项目代码	结果	单位	参考范围
肌酸激酶	CK	1281 ↑	U/L	25-190	总胆汁酸	TBA	8.1	umol/L	0-10
肌酸激酶同工酶	CKMB	49 ↑	U/L	0-25	钾	K+	3.36 ↓	mmol/L	3.5-5.3
a-羟丁酸脱氢酶	HBDH	395 ↑	U/L	72-182	钠	Na+	128.6 ↓	mmol/L	137-147
乳酸脱氢酶	LDH	430 ↑	U/L	120-250	氯	CL-	94.9 ↓	mmol/L	99-110
谷丙转氨酶	ALT	45	U/L	9-50	钙	TCa	2.10 ↓	mmol/L	2.11-2.52
谷草转氨酶	AST	126 ↑	U/L	15-40	离子钙	iCa	1.08 ↓	mmol/L/L	1.1-1.35
总蛋白	TP	47.6 ↓	g/L	60-85	尿素	UREA	4.51	mmol/L	3.6-9.5
白蛋白	ALB	26.9 ↓	g/L	35-55	肌酐	CREA	44.3 ↓	umol/L	57-111
球蛋白	GLO	20.7	g/L	15-40	尿酸	UAC	221	umol/L	208-428
前白蛋白	PA	104.9 ↓	mg/L	200-400	β2-微球蛋白	β2-mG	3.4 ↑	mg/L	1-3
总胆红素	TBIL	15.8	umol/L	3.4-25					
直接胆红素	DBIL	10.0 ↑	umol/L	0-7					
间接胆红素	IBil	5.8	umol/L	0-21					
碱性磷酸酶	ALKP	63	U/L	45-125					
谷氨酰转肽酶	GGT	27	U/L	10-60					
胆碱酯酶	CHE	2478 ↓	U/L	4000-12600					

采样时间：　签收时间：2024-05-15 15:45　审核时间：2024-05-15 17:09
检 验 者：　审 核 者：
本检测结果仅对来样负责，如有疑议请在报告时间后7天内与我们联系，谢谢合作！

Supplemental Fig.4. Serological testing revealed markedly elevated creatine kinase (CK) levels.

Supplemental Fig.5

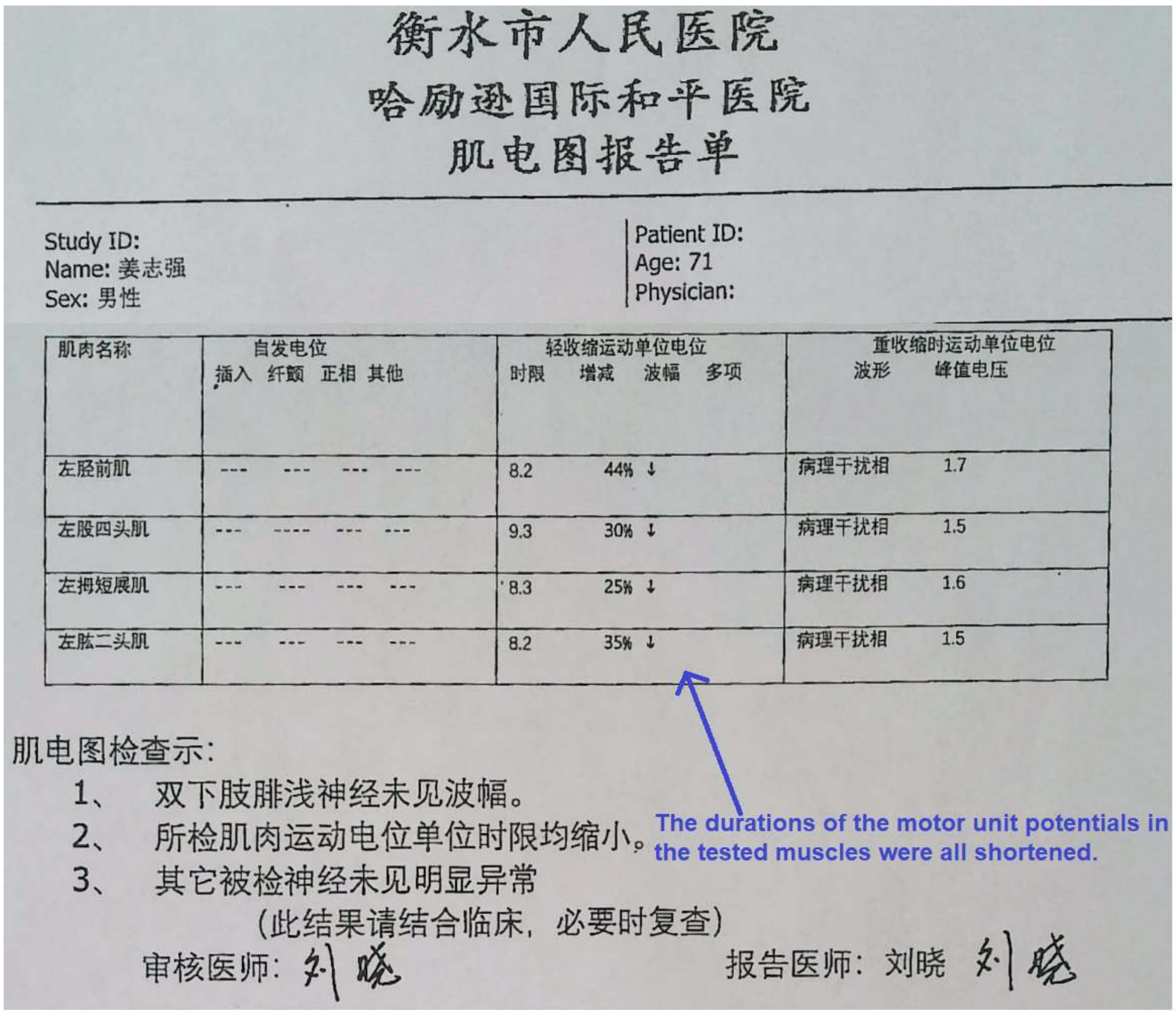

衡水市人民医院
哈励逊国际和平医院
肌电图报告单

Study ID:
Name: 姜志强
Sex: 男性
Patient ID:
Age: 71
Physician:

肌肉名称	自发电位				轻收缩运动单位电位				重收缩时运动单位电位	
	插入	纤颤	正相	其他	时限	增减	波幅	多项	波形	峰值电压
左胫前肌	---	---	---	---	8.2	44% ↓			病理干扰相	1.7
左股四头肌	---	----	---	---	9.3	30% ↓			病理干扰相	1.5
左拇短展肌	---	---	---	---	8.3	25% ↓			病理干扰相	1.6
左肱二头肌	---	---	---	---	8.2	35% ↓			病理干扰相	1.5

肌电图检查示:
1、 双下肢腓浅神经未见波幅。
2、 所检肌肉运动电位单位时限均缩小。
3、 其它被检神经未见明显异常
(此结果请结合临床，必要时复查)
审核医师:　报告医师: 刘晓

Supplemental Fig.5: Electromyography (EMG) showed that the motor unit potential durations in all tested muscles were shortened.

Supplemental Fig.6

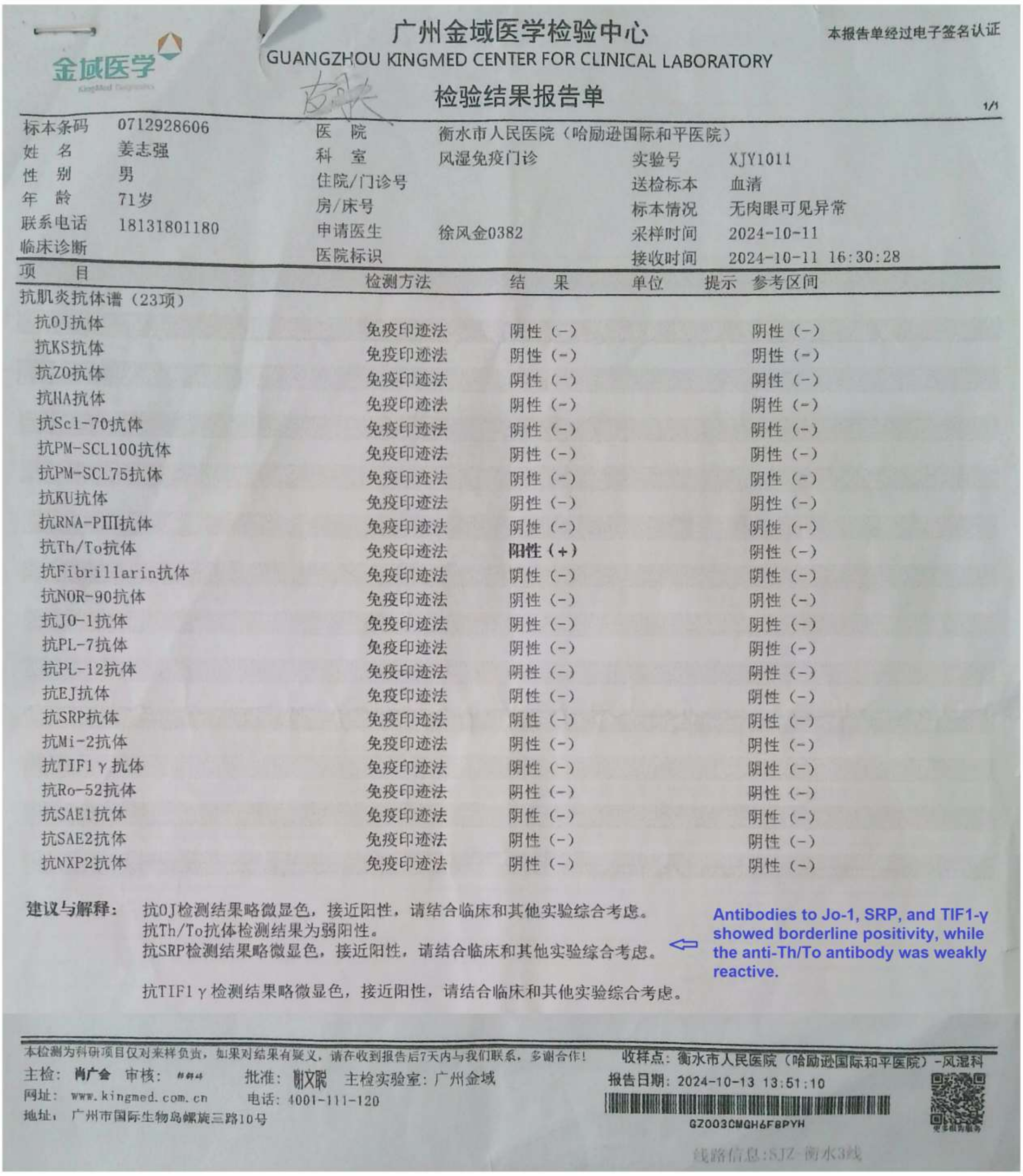

广州金域医学检验中心
GUANGZHOU KINGMED CENTER FOR CLINICAL LABORATORY
检验结果报告单

金域医学
本报告单经过电子签名认证
1/1

标本条码 0712928606
姓 名 姜志强
性 别 男
年 龄 71岁
联系电话 18131801180
临床诊断

医 院 衡水市人民医院（哈励逊国际和平医院）
科 室 风湿免疫门诊
住院/门诊号
房/床号
申请医生 徐风金0382
医院标识

实验号 XJY1011
送检标本 血清
标本情况 无肉眼可见异常
采样时间 2024-10-11
接收时间 2024-10-11 16:30:28

项 目	检测方法	结 果	单位	提示	参考区间
抗肌炎抗体谱（23项）					
抗OJ抗体	免疫印迹法	阴性（-）			阴性（-）
抗KS抗体	免疫印迹法	阴性（-）			阴性（-）
抗ZO抗体	免疫印迹法	阴性（-）			阴性（-）
抗HA抗体	免疫印迹法	阴性（-）			阴性（-）
抗Scl-70抗体	免疫印迹法	阴性（-）			阴性（-）
抗PM-SCL100抗体	免疫印迹法	阴性（-）			阴性（-）
抗PM-SCL75抗体	免疫印迹法	阴性（-）			阴性（-）
抗KU抗体	免疫印迹法	阴性（-）			阴性（-）
抗RNA-PIII抗体	免疫印迹法	阴性（-）			阴性（-）
抗Th/To抗体	免疫印迹法	**阳性（+）**			阴性（-）
抗Fibrillarin抗体	免疫印迹法	阴性（-）			阴性（-）
抗NOR-90抗体	免疫印迹法	阴性（-）			阴性（-）
抗JO-1抗体	免疫印迹法	阴性（-）			阴性（-）
抗PL-7抗体	免疫印迹法	阴性（-）			阴性（-）
抗PL-12抗体	免疫印迹法	阴性（-）			阴性（-）
抗EJ抗体	免疫印迹法	阴性（-）			阴性（-）
抗SRP抗体	免疫印迹法	阴性（-）			阴性（-）
抗Mi-2抗体	免疫印迹法	阴性（-）			阴性（-）
抗TIF1γ抗体	免疫印迹法	阴性（-）			阴性（-）
抗Ro-52抗体	免疫印迹法	阴性（-）			阴性（-）
抗SAE1抗体	免疫印迹法	阴性（-）			阴性（-）
抗SAE2抗体	免疫印迹法	阴性（-）			阴性（-）
抗NXP2抗体	免疫印迹法	阴性（-）			阴性（-）

建议与解释： 抗OJ检测结果略微显色，接近阳性，请结合临床和其他实验综合考虑。
抗Th/To抗体检测结果为弱阳性。
抗SRP检测结果略微显色，接近阳性，请结合临床和其他实验综合考虑。
抗TIF1γ检测结果略微显色，接近阳性，请结合临床和其他实验综合考虑。

Antibodies to Jo-1, SRP, and TIF1-γ showed borderline positivity, while the anti-Th/To antibody was weakly reactive.

本检测为科研项目仅对来样负责，如果对结果有疑义，请在收到报告后7天内与我们联系，多谢合作！
主检： 尚广金 审核：
批准： 谢文熙 主检实验室：广州金域
网址： www.kingmed.com.cn 电话： 4001-111-120
地址： 广州市国际生物岛螺旋三路10号
收样点：衡水市人民医院（哈励逊国际和平医院）-风湿科
报告日期：2024-10-13 13:51:10
GZ003CMQH6F8PYH
线路信息：SJZ-衡水3线

Supplemental Fig.6: Borderline positivity was observed for anti-Jo-1, anti-SRP, and anti-TIF1γ, whereas anti-Th/To was weakly reactive.

Supplemental Fig.7

项目名称:肿瘤标志物组合2,

北京高博医院检验报告单

标本号:8

姓名:姜志强	性别:男	年龄:72岁	ID号:10021862
科室:综合内科	床号:11	送检医生:刘桂霞	住院号:003271
标本类型:血	送检单位:	检测仪器:罗氏E602	条码号:2251118475433
申请时间:2025-11-18 15:55:10	采样时间:2025-11-18 16:37:19	临床诊断:恶性肿瘤放疗	

序号	代号	项目名称	结果		单位	参考范围	检测方法
1	AFP	甲胎蛋白	1.71		ng/mL	0-7	
2	CEA	癌胚抗原	160.00	↑	ng/mL	0-5	
3	NSE	神经元特异性烯醇化酶测定	362.20	↑	ng/mL	0-16.3	
4	CYFRA21-1	细胞角蛋白19片段测定	52.47	↑	ng/mL	0-3.3	
5	SCC	鳞状上皮细胞癌抗原	3.12	↑	ng/mL	0-3	
6	ProGRP	胃泌素释放肽前体	11278.39	↑	pg/ml	0-65.7	

接收时间:2025-11-18 16:37:33　报告时间:2025-11-19 10:45:41　检验者:李[illegible]秋　审核者:

本报告仅对此次送检样本负责　实验室地址：北京市昌平区科学园路4号院1号楼，联系电话010-50847588　第1页 共1页

备注:

项目名称:肝功组合,离子组合2,

北京高博医院检验报告单

标本号:1018

姓名:姜志强	性别:男	年龄:72岁	ID号:10021862
科室:综合内科	床号:11	送检医生:刘桂霞	住院号:003271
标本类型:静脉血清	送检单位:	检测仪器:日立008	条码号:2251119475898
申请时间:2025-11-19 09:36:52	采样时间:2025-11-20 05:22:28	临床诊断:恶性肿瘤放疗	

	英文	名称	结果		参考范围	单位
1	ALT	丙氨酸氨基转移酶	61.00	↑	9-50	U/L
2	AST	天门冬氨酸氨基转	276.00	↑	15-40	U/L
3	TP	总蛋白	66.80		65-85	g/L
4	Alb	白蛋白(溴甲酚绿法	31.00	↓	40-55	g/L
5	GLOB	球蛋白	35.80		20-40	g/L
6	A/G	白球比	0.87	↓	(1.2-2.4):1	
7	PA	前白蛋白	40.00	↓	200-430	mg/L
8	TBil	总胆红素	338.30	↑	0-26	umol/L
9	DBil	直接胆红素	267.00	↑	0-6.8	umol/L
10	IBil	间接胆红素	71.30	↑	2-15.22	umol/L
11	TBA	总胆汁酸	259.20	↑	0-10	umol/L
12	ALP	碱性磷酸酶	286.00	↑	45-125	U/L
13	GGT	γ-谷氨酰基转移酶	177.00	↑	10-60	U/L
14	ChE	胆碱酯酶	1623	↓	4620-11500	U/L
15	LDH	乳酸脱氢酶	517.00	↑	120-250	U/L
16	CK	肌酸激酶	216.00		50-310	U/L
17	CK-MB	肌酸激酶同工酶（	317.00	↑	0-24	U/L
18	K	钾	4.08		3.5-5.3	mmol/L
19	Na	钠	143.90		137-147	mmol/L
20	Cl	氯	112.50	↑	99-110	mmol/L

接收时间:2025-11-20 06:00:12　报告时间:2025-11-20 10:11:19　检验者:李[illegible]秋　审核者:

Supplemental Fig.7: Serum levels of LDH, NSE, and ProGRP were significantly elevated, which supported the diagnosis of a malignant tumor.
