# Supplementary material for: Paraneoplastic dermatomyositis-like syndrome preceding diagnosis of small cell lung cancer: a case report
Source: Front Oncol. 2026 Jul 31;16:1915555. doi: 10.3389/fonc.2026.1915555 (PMC13472917; doi:10.3389/fonc.2026.1915555)
Supplement: Supplementary file 2 [file Table1.docx]

**Supplemental data**

Supplemental Table 1

| Time line | Events | Remarks |
| --- | --- | --- |
| 2021 | Digital clubbing | Initial paraneoplastic manifestation |
| 2023 | Skin rash | Progressive cutaneous involvement |
| Apr 2024 | Forehead skin biopsy (pathology & IHC) | Erythroderma (Interface dermatitis with mucin deposition & mixed lymphocytic infiltration) |
| Apr 2024 | PET/CT | Lung mass detected ( Extensive-stage ) |
| Apr 2024 | Tumor biopsy  (pathology & IHC) | SCLC |
| May 2024 | Serological and EMG tests | DM‑like changes |
| May 2024 - Oct 2024 | First‑line chemotherapy | EC |
| Follow‑up | Imaging | Confirmed PR on follow‑up imaging |
|  | Clinical | Resolution of DM- like features (Fig.1c) |
| Apr 2025 - May 2025 | Switch to second‑line (nab‑paclitaxel monotherapy) | Due to the progression of the tumor and skin rash |
| Follow‑up | Imaging | Brief complete remission (~3 months) (Fig.2c) then progression from Aug. 2025 onward |
|  | Clinical | Resolution of skin rash, then progression; died of hepatic metastases and liver failure in Nov. 2025 |

**Supplemental Table 1. Clinical timeline of the patient‘s disease course.** This timeline illustrates the sequential clinical events from initial paraneoplastic manifestations to diagnosis, treatment, and final outcome.

Abbreviations: SCLC, small cell lung carcinoma; DM, dermatomyositis; EMG, electromyography; CK, creatine kinase; PR, partial response; CR, complete response; EC, etoposide plus carboplatin.

**Supplemental Fig.1**

Supplemental Fig.1**:**Forehead skin biopsy revealed mild epidermal atrophy, scattered apoptotic cells, multifocal basal cell degeneration, dermal edema, and increased mucin deposition, consistent with interface dermatitis, perivascular lymphocytic infiltration, and mucin deposition. IHC showed a mixed lymphocytic infiltrate (CD3+, CD4+, CD8+, focally CD20+, CD163+)

**Supplemental Fig.2**

Supplemental Fig.2: Diagnosis of extensive-stage of lung cancer.

**Supplemental Fig.3**

Supplemental Fig.3: Diagnosis of SCLC by Immunohistochemistry : CK (pancytokeratin cocktail) diffuse positive (+); CgA(+); KI-67(80%+); TIF-1 (diffuse +); INSM1 (majority +); P40(-); CK5/6 (occasional positive); NapsinA(-); NUT(-); BRG1(diffuse +)
**Supplemental Fig.4**

Supplemental Fig.4. Serological testing revealed markedly elevated creatine kinase (CK) levels.

**Supplemental Fig.5**

Supplemental Fig.5: Electromyography (EMG) showed that the motor unit potential durations in all tested muscles were shortened.
**Supplemental Fig.6**

Supplemental Fig.6: Borderline positivity was observed for anti‑Jo‑1, anti‑SRP, and anti‑TIF1γ, whereas anti‑Th/To was weakly reactive.

**Supplemental Fig.7**

Supplemental Fig.7: Serum levels of LDH, NSE, and ProGRP were significantly elevated, which supported the diagnosis of a malignant tumor.
